# Prior exposure to alkylating agents negatively impacts testicular organoid formation in cells obtained from childhood cancer patients

**DOI:** 10.1093/hropen/hoae049

**Published:** 2024-08-13

**Authors:** Yanhua Cui, Femke Harteveld, Hajar Ali Mohammed Ba Omar, Yifan Yang, Ragnar Bjarnason, Patrik Romerius, Mikael Sundin, Ulrika Norén Nyström, Cecilia Langenskiöld, Hartmut Vogt, Lars Henningsohn, Per Frisk, Kaisa Vepsäläinen, Cecilia Petersen, Rod T Mitchell, Jingtao Guo, João Pedro Alves-Lopes, Kirsi Jahnukainen, Jan-Bernd Stukenborg

**Affiliations:** NORDFERTIL Research Lab Stockholm, Childhood Cancer Research Unit, Department of Women’s and Children’s Health, Karolinska Institutet, Karolinska University Hospital, Solna, Sweden; NORDFERTIL Research Lab Stockholm, Childhood Cancer Research Unit, Department of Women’s and Children’s Health, Karolinska Institutet, Karolinska University Hospital, Solna, Sweden; NORDFERTIL Research Lab Stockholm, Childhood Cancer Research Unit, Department of Women’s and Children’s Health, Karolinska Institutet, Karolinska University Hospital, Solna, Sweden; NORDFERTIL Research Lab Stockholm, Childhood Cancer Research Unit, Department of Women’s and Children’s Health, Karolinska Institutet, Karolinska University Hospital, Solna, Sweden; Children’s Medical Center, Landspítali University Hospital, Reykjavik, Iceland; Department of Paediatrics, Faculty of Medicine, University of Iceland, Reykjavik, Iceland; Department of Paediatric Oncology and Haematology, Clinical Sciences, Lund University, Barn-och Ungdomssjukhuset Lund, Skånes Universitetssjukhus, Lund, Sweden; Division of Paediatrics, Department of Clinical Science, Intervention and Technology, Karolinska Institutet, Huddinge, Sweden; Pediatric Blood Disorders, Immunodeficiency and Stem Cell Transplantation Unit, Astrid Lindgren Children’s Hospital, Karolinska University Hospital, Huddinge, Sweden; Department of Clinical Sciences, Pediatrics, Umeå University, Umeå, Sweden; Department of Paediatric Oncology, The Queen Silvia Children’s Hospital, Gothenburg, Sweden; Department of Biomedical and Clinical Science, H.R.H Crown Princess Victoria’s Children’s and Youth Hospital, Linköping University and University Hospital, Linköping, Sweden; Division of Urology, Department of Clinical Science, Intervention and Technology, Karolinska Institutet, Huddinge, Sweden; Pediatric Hematology & Oncology, Children’s University Hospital, Uppsala, Sweden; Department of Paediatrics, Kuopio University Hospital, Kuopio, Finland; NORDFERTIL Research Lab Stockholm, Childhood Cancer Research Unit, Department of Women’s and Children’s Health, Karolinska Institutet, Karolinska University Hospital, Solna, Sweden; MRC Centre for Reproductive Health, Institute for Regeneration and Repair, Edinburgh BioQuarter, The University of Edinburgh, Edinburgh, UK; Royal Hospital for Children and Young People, Edinburgh, UK; State Key Laboratory of Stem Cell and Reproductive Biology, Institute of Zoology, Chinese Academy of Sciences, Beijing, China; Beijing Institute for Stem Cell and Regenerative Medicine, Beijing, China; University of Chinese Academy of Sciences, Beijing, China; NORDFERTIL Research Lab Stockholm, Childhood Cancer Research Unit, Department of Women’s and Children’s Health, Karolinska Institutet, Karolinska University Hospital, Solna, Sweden; NORDFERTIL Research Lab Stockholm, Childhood Cancer Research Unit, Department of Women’s and Children’s Health, Karolinska Institutet, Karolinska University Hospital, Solna, Sweden; New Children’s Hospital, Paediatric Research Centre, Department of Paediatrics, University of Helsinki and Helsinki University Hospital, Helsinki, Finland; NORDFERTIL Research Lab Stockholm, Childhood Cancer Research Unit, Department of Women’s and Children’s Health, Karolinska Institutet, Karolinska University Hospital, Solna, Sweden

**Keywords:** cell culture, fertility preservation, germ cells, testis, tissue engineering, organoids, childhood cancer, chemotherapy, late effects

## Abstract

**STUDY QUESTION:**

Can human pre- and peri-pubertal testicular cells obtained from childhood cancer patients, previously treated with chemotherapy, form testicular organoids (TOs)?

**SUMMARY ANSWER:**

Organoid formation from testicular tissue collected from childhood cancer patients positively correlates with SRY-Box transcription factor 9 (SOX9) expression in Sertoli cells, which in turn negatively correlates with previous exposure to alkylating chemotherapy.

**WHAT IS KNOWN ALREADY:**

Pre- and peri-pubertal boys exposed to highly gonadotoxic therapies can only safeguard their fertility potential through testicular tissue cryopreservation. Today, there is no established clinical tool to restore fertility using these testicular samples. Organoids hold promise in providing fundamental early insights in creating such platforms. However, the generation of TOs that closely resemble the innate testis, to enable a thorough monitoring of the necessary steps for germ cell differentiation and somatic functionalities, remains a challenge.

**STUDY DESIGN, SIZE, DURATION:**

We used a Matrigel-based three-layer gradient culture system to generate human TOs and to reveal whether chemotherapy exposure affects TO formation capacity and the functionality of pre- and peri-pubertal testicular somatic cells. Testicular cells of 11 boys (aged 7.7 ± 4.1 (mean ± SD) years) were assessed for TO formation in relation to previous chemotherapy exposure and SOX9 expression in histological sections of paraffin-embedded testicular tissue samples collected on the day of biopsy and compared with testicular tissue samples obtained from 28 consecutive patients (aged 6.9 ± 3.8 (mean ± SD) years). All 39 patients were part of the fertility preservation project NORDFERTIL; an additional 10 samples (from boys aged 5.5 ± 3.5 (mean ± SD) years, without an underlying pathology) in an internal biobank collection were used as controls.

**PARTICIPANTS/MATERIALS, SETTING, METHODS:**

We obtained 49 testicular tissue samples from boys aged 0.8–13.4 years. Fresh samples (n = 11) were dissociated into single-cell suspensions and applied to a three-layer gradient culture system for organoid formation. Histological sections of another 28 samples obtained as part of the fertility preservation project NORDFERTIL, and 10 samples from a sample collection of a pathology biobank were used to evaluate the effects of prior exposure to alkylating agents on testicular samples. Testicular organoid formation was defined based on morphological features, such as compartmentalized structures showing cord formation, and protein expression of testicular cell-specific markers for germ and somatic cells was evaluated via immunohistochemical staining. Hormone secretion was analysed by specific enzyme-linked immunosorbent assays for testosterone and anti-Müllerian hormone (AMH) production.

**MAIN RESULTS AND THE ROLE OF CHANCE:**

Our results revealed that 4 out of 11 prepubertal testicular samples formed TOs that showed compartmentalized cord-like structures surrounded by interstitial-like areas and increasing levels of both testosterone as well as AMH over a 7-day culture period. We observed that SOX9 expression was correlated positively with TO formation. Moreover, exposure to alkylating agents before biopsy was inversely correlated with SOX9 expression (*P* = 0.006).

**LARGE SCALE DATA:**

N/A.

**LIMITATIONS, REASONS FOR CAUTION:**

Due to the limited amount of material available, only 11 out of the 39 pre- and peri-pubertal testicular tissue samples could be used for the organoid formation experiments. The testicular tissue samples obtained from a sample collection of the internal biobank of Department of Pathology, Karolinska University Hospital were considered normal and included in the study if no testicular pathology was reported. However, detailed information regarding previous medical treatments and/or testicular volumes of the patients included in this biobank was not available.

**WIDER IMPLICATIONS OF THE FINDINGS:**

Our observations suggest that SOX9 expression may serve as a putative indicator of TO formation, indicating a critical role of Sertoli cells in promoting organoid formation, seminiferous tubule integrity, and testicular function in pre- and peri-pubertal testicular tissue.

**STUDY FUNDING/COMPETING INTEREST(S):**

This study was supported by grants from the Swedish Childhood Cancer Foundation (PR2019-0123; PR2022-0115; TJ2020-0023) (J.-B.S.), Finnish Cancer Society (K.J.), Finnish Foundation for Paediatric Research (K.J.), Swedish Research Council (2018-03094; 2021-02107) (J.-B.S.), and Birgitta and Carl-Axel Rydbeck’s Research Grant for Paediatric Research (2020-00348; 2020-00335; 2021-00073; 2022-00317) (J.-B.S. and K.J.). Y.C. and Y.Y. received a scholarship from the Chinese Scholarship Council. J.P.A-L. was supported by a Starting Grant in Medicine and Health (2022-01467) from the Swedish Research Council. R.T.M. was supported by a UKRI Future Leaders Fellowship (MR/S017151/1). The MRC Centre for Reproductive Health was supported by an MRC Centre Grant (MR/N022556/1). The authors declare no competing interests.

WHAT DOES THIS MEAN FOR PATIENTS? Treatments for cancer in boys can harm their testicles and lead to problems with fertility later in life. As more children survive cancer, maintaining their ability to have children in the future has become very important. Scientists have shown that with samples from monkeys, freezing and later re-implanting immature testicular tissue can lead to sperm production and healthy offspring. However, this technique is still experimental in humans and cannot be used for all patients, especially those whose testicular tissue might contain cancer cells.

To find alternative methods, we studied the testicular cells from boys who had cancer to see if they could form ‘mini testicles’ called ‘testicular organoids’ in a laboratory setting. We also looked at how past chemotherapy treatments affected this process. We found that a specific somatic cell marker, SRY-Box transcription factor 9 (SOX9), was linked to the ability of the cells to form these ‘mini testicles’. Higher levels of SOX9 meant better formation of testicular organoids, while exposure to certain chemotherapy drugs and doses reduced the SOX9 levels.

Our research suggests that measuring SOX9, along with hormone levels, could help evaluate the quality of testicular tissue from male childhood cancer patients. Additionally, these lab-grown ‘mini testicles’ could be a new way to study how testicles develop, how sperm stem cells function, and the effects of genetic diseases and drugs. This knowledge could improve how we use frozen testicular tissue to help cancer survivors have children in the future.

## Introduction

Cancer therapies and conditioning regimens for haematopoietic stem cell transplantation in childhood can negatively affect testicular function and lead to infertility. With the increasing number of long-term childhood cancer survivors, fertility preservation has become a high priority for these patients and their families (Jahnukainen *et al.*, 2015; Goossens *et al.*, 2020; Duffin *et al.*, 2024). Proof-of-concept studies in non-human primates following autotransplantation of cryopreserved immature testicular tissue have resulted in full spermatogenesis and the generation of live offspring (Fayomi *et al.*, 2019). However, this procedure is still experimental in humans and is not applicable to every patient group, for example, in patients at risk of malignant infiltration in their testicular tissue samples. Therefore, additional strategies are required. One alternative option is to establish *in vitro* spermatogenesis from immature testicular tissue preserved prior to treatment (Giudice *et al.*, 2017; Lei *et al.*, 2023). Successful generation of haploid germ cells, first demonstrated in mice (Sato *et al.*, 2011), has been reported in various animal models, including humans (Yang *et al.*, 2014; Reda *et al.*, 2016, 2017; de Michele *et al.*, 2018). However, results showing functional sperm in humans are still lacking, mainly due to limitations of the current *in vitro* culture conditions. Therefore, modelling testicular architecture and the spermatogonial stem cell (SSC) niche using testicular organoids (TOs) offer a promising platform to gain insights on the *in vitro* conditions necessary for restoring fertility in boys (Alves-Lopes and Stukenborg, 2018; Oliver and Stukenborg, 2020; Haider and Beristain, 2023).

Sertoli cells play a crucial role in establishing the physical framework for the SSC niche, driving germ cell differentiation and regulating testicular function. During human embryonic development, early Sertoli cells are specified from bipotent early supporting gonadal cells ∼7–8 weeks after conception (Garcia-Alonso *et al.*, 2022). Specification of the early Sertoli cell fate in the immature human gonads is triggered by the sequential activation of the sex-determining region of the Y chromosome (SRY) and its downstream target gene, SRY-Box transcription factor 9 (SOX9) (Hanley *et al.*, 2000; Ming *et al.*, 2022). During subsequent development of the embryonic and foetal testis, the expression of SOX9 remains vital to promote early Sertoli cell maturation and formation of testicular cords, essential for the establishment of the SSC niche (Ming *et al.*, 2022). Apart from their key role during pre-natal development, Sertoli cells are crucial for maturation events occurring in the testis during puberty and adulthood, reflected by the dynamic transcriptomic changes experienced by these cells during this period (Guo *et al.*, 2020). Specifically, Sertoli cells establish intercellular connections to form the blood–testis barrier, which is essential for the structural integrity of the seminiferous tubules and the establishment of the SSC niche (O'Donnell *et al.*, 2022). In the adult testis, Sertoli cells will sustain spermatogenesis and their total number will set the testicular volume and sperm production (Johnson *et al.*, 1984).

Previously, we described the successful formation of TOs from rodent post-natal and human pre-natal testicular tissue samples (Alves-Lopes *et al.*, 2017, 2018; Oliver *et al.*, 2021). However, the capacity of human pre- and peri-pubertal testicular cells to form TOs has not yet been tested. Moreover, the impact of previous cancer therapy in TOs formation remains unclear.

In this study, we therefore investigated the organoid formation capacity of human pre- and peri-pubertal testicular cells obtained from childhood cancer patients and assessed the impact of previous chemotherapy exposure on organoid formation. Importantly, we observed that SOX9 expression in Sertoli cells was positively correlated with the capacity to form organoids and negatively correlated with previous exposure to alkylating agents in testicular samples collected for fertility preservation from childhood cancer patients.

## Materials and methods

### Ethical approval

Ethical approval for the use of testicular tissues was obtained from the Regional Ethics Board in Stockholm (archived sample collection of the Department of Pathology at Karolinska University Hospital: Dnr 2014-267-31/4, and Dnr 2024-01273-02; NORDFERTIL sample collection: Dnr 2013-2129-31-3, and Dnr 2021-04277), National Ethics Board of Iceland, Reykjavik (VSN 15-002) and Ethics Board of the University of Helsinki (426/13/03/03/2015).

### Sample collection

Testicular biopsy samples were obtained from pre- and peri-pubertal patients (n = 49; age range from 0.8 to 13.4 years; identified as P1–49, Supplementary Table S1). Sections from 10 paraffin-embedded testicular tissue samples, obtained from the biobank at the Department of Pathology at Karolinska University Hospital, without underlying pathologies served as controls. The remaining 39 samples were obtained from patients who participated in the NORDFERTIL fertility preservation project (NORDFERTIL sample collection). The inclusion criteria for the study was a very high risk of subfertility (>80%) due to the planned treatments (allogenic/autologous haematopoietic stem cell transplantation or radiotherapy involving the testis), based on available evidence from the literature (Skinner *et al.*, 2017). Exclusion criteria were a high risk of bleeding and/or infection, and a testicular volume >10 ml (measured by orchidometer). The basic characteristics of the 39 patients, including age, diagnosis, and cancer therapy prior to testicular biopsy, were reported by their designated physicians (Supplementary Tables S1 and S2). Alkylating agent exposure was calculated as the mean cumulative cyclophosphamide equivalent dose (CED) (Green *et al.*, 2014) and anthracycline exposure was calculated as cumulative doxorubicin isotoxic dose equivalents (DIE) using a conversion factor of 1.0 for doxorubicin, 0.67 for epirubicin, 5.0 for idarubicin, and 4.0 for mitoxantrone (Shankar *et al.*, 2008) as well as 0.6 for daunorubicin (Feijen *et al.*, 2019) (Supplementary Table S1). Verbal and written consent for participation in the research project was obtained from the parents and patients for all samples. To preserve fertility, 20% of the testicular volume of one testis was obtained. Two-thirds of this 20% were cryopreserved for future clinical fertility preservation; the remaining one-third were transported as fresh tissue to the NORDFERTIL research laboratory at Karolinska Institutet for research and was included in this study. Approximately 5% of this fresh testicular tissue was immediately fixed in 4% paraformaldehyde (PFA, HL96753.1000, Histolab, Askim, Sweden) and embedded in paraffin for histological analysis, whereas the remaining fresh tissue was used for tissue and cell culture experiments. Of the 39 samples utilized for tissue and cell culture experiments, 11 (P1–P11) consecutive (unselected) samples with enough material for single-cell preparation, were tested for the TO cultures described in this publication.

### Three-layer gradient system

**Figure 1.**
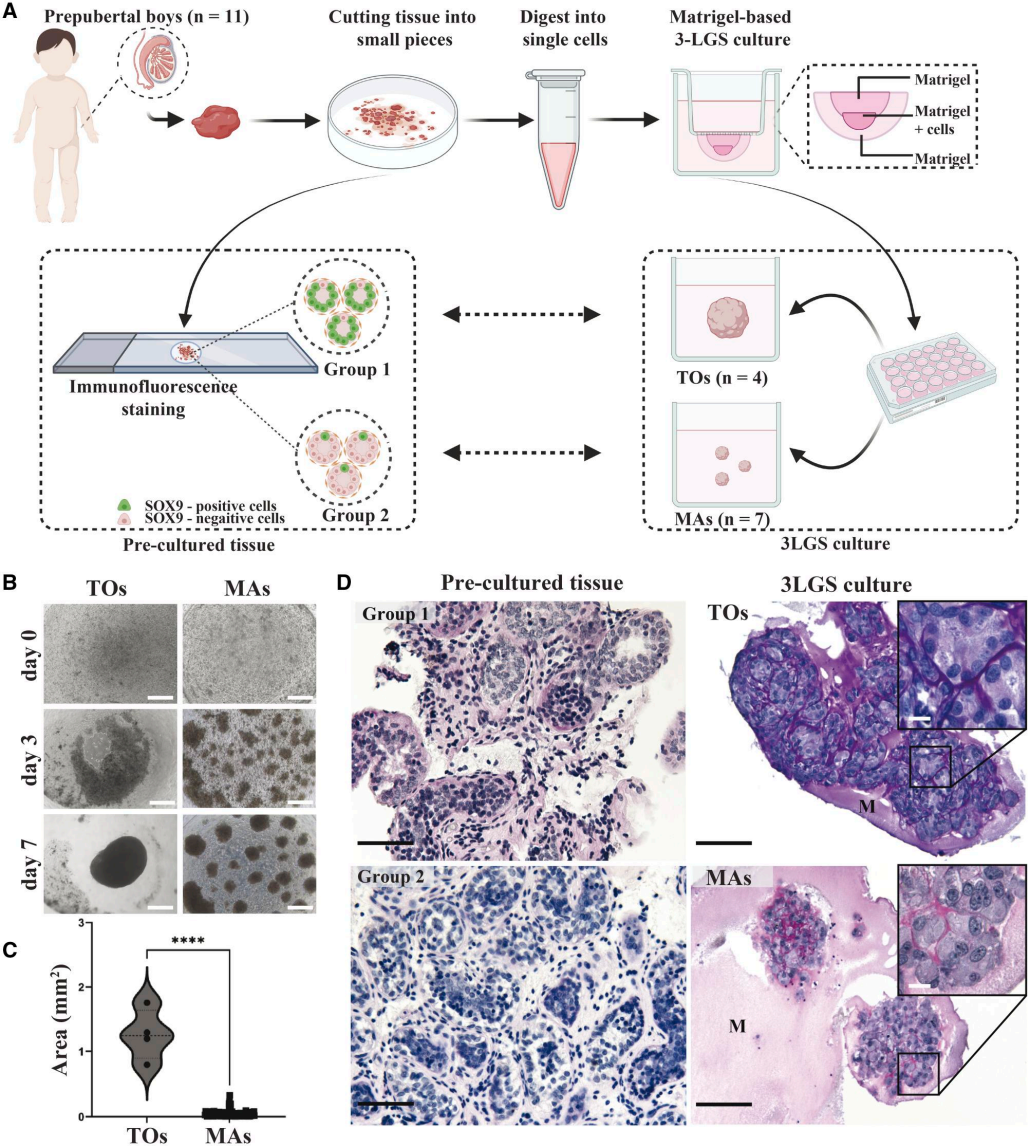
**Generation of human prepubertal testicular organoids (TOs)**. (**A**) Schematic illustration of the study design (created with BioRender^®^). (**B**) Representative images of the human prepubertal testicular cells reorganizing into single aggregates (posteriorly classified as TOs) and multiple aggregates (MAs) during 7 days of culture. Scale bars = 500 µm. (**C**) Analysis of the area of single aggregates (subsequently classified as TOs) and MAs. The graph shows the area of all individual aggregates formed after 7 days of culture. The Mann–Whitney test was used to determine the statistical significance between the groups. ****P < 0.0001. (**D**) Representative PAS staining images showing the histology of testicular samples of patients from Groups 1 and 2 (left panel) and cord-like structures and interstitial-like compartments in TOs, as well as the lack of similar structures in MAs (right panel). M, Matrigel; SOX9, SRY-Box transcription factor 9; 3LGS, three-layer gradient system; PAS, periodic acid–Schiff. Scale bars = 100 µm (insets, 10 µm).

Small tissue fragments (∼1 mm^3^) of pre- and peri-pubertal testicular tissue samples (n = 11; 0.8–13.2 years of age) were enzymatically digested into a cell suspension of primary testicular cells including all testicular somatic and germ cells. This cell suspension was applied to the three-layer gradient system (3-LGS), which consists of a layer of testicular cells suspended in Matrigel placed between two cell-free Matrigel layers (Fig. 1A) (Alves-Lopes *et al.*, 2018). TOs were cultured in NutriStem (05-100-1, Biological Industries, Kibbutz Beit-Haemek, Israel) with 10% KSR (KnockOut serum replacement XenoFree; 10828-028, Gibco, Grand Island, NY, USA), 1% penicillin and streptomycin (15070-063, Gibco) and 10^−7^ M melatonin (M5250-1G, Sigma-Aldrich, St. Louis, MO, USA) in a 24-well plate (734-2325, VWR, Stockholm, Sweden), for 7 days at 35°C, with medium changes every 48 h. For each patient, at least three technical replicates were conducted.

### Measuring organoid formation

Representative bright-field images (40-fold magnification using 4× objective lenses) were acquired every day to measure the area of the generated structures (organoids or aggregates). The total area was automatically measured using Fiji/ImageJ software (U.S. National Institutes of Health, Bethesda, MD, USA) using particle analysis in default settings.

### Periodic acid–Schiff staining

TOs and tissue fragments were fixed in 4% PFA (HL96753.1000, Histolab) or Bouin’s solution (Sigma-Aldrich, HT10132). After dehydration, the samples were embedded in paraffin (Paraplast, P3808, Sigma-Aldrich) and cut into 5-µm thick sections using a microtome (HM350, Microm, Heidelberg, Germany). For morphological analysis, paraffin-embedded organoids and tissue fragments were stained with a periodic acid–Schiff staining kit (101646, Sigma-Aldrich). Briefly, the sections were deparaffinized in xylene (1330-20-7, Fisher Scientific, Hampton, NH, USA) and rehydrated in a descending ethanol series: 100% ethanol (01399.01 l, Histolab), 96% ethanol (01396.01 l, HistoLab), and 70% ethanol (01370.01 l, HistoLab). The sections were then oxidized in 0.5% periodic acid solution for 5 min, incubated in Schiff’s reagent for 15 min, and counterstained with haematoxylin solution (105175, Sigma-Aldrich) for 1 min. Between each step, sections were rinsed with distilled water. Finally, the sections were dehydrated and mounted using the xylene-based mounting agent Pertex (1721183, Histolab). Images were obtained using a bright-field microscope (DFC290, Leica, Wetzlar, Germany).

### Immunofluorescence staining

For immunofluorescence analysis, three sections per sample were subjected to heat-mediated antigen retrieval at 95°C in 10 mM sodium citrate (C7254, Sigma-Aldrich) at a pH of 6 for 20 min. Subsequently, the samples were blocked in tris-buffered saline (TBS) supplemented with 10% normal donkey serum (017-000-121, Jackson Immuno Research, West Grove, PA, USA) and 1% bovine serum albumin (A2153, Sigma-Aldrich). The primary antibodies against DEAD-box helicase 4 (DDX4), SOX9, Wilms tumour protein 1 (WT1), anti-Müllerian hormone (AMH), GATA binding protein 1 (GATA1) androgen receptor (AR), cytochrome P450 family 17 subfamily A member 1 (CYP17A1), actin alpha 2 (ACTA2), and laminin alpha 1 (LAMA1, extracellular matrix) (Supplementary Table S3) were diluted in blocking buffer, and the sections were incubated overnight at 4°C. Mouse or rabbit IgGs were used as negative controls according to the host of the primary antibody used (Supplementary Table S3). After three washing steps in TBS for 5 min each, the samples were incubated at room temperature with fluorescent-conjugated secondary antibodies (Supplementary Table S3), and 4′,6-diamidino-2-phenylindole (DAPI) (135-1303, Bio-Rad, Hercules, CA, USA), which were diluted in blocking buffer. The slides were mounted with an anti-fade mounting medium (P36931, Invitrogen, Waltham, MA, USA). Immunofluorescence images were obtained using a confocal microscope (LSM700, Zeiss, Jena, Germany).

### Evaluation of immunofluorescence staining

The intensity of fluorescence can vary across samples due to factors like antibody binding efficiency, fluorochrome brightness, and sample preparation. This variability can affect accurate quantification. To ensure consistent quantification, WT1-, SOX9-, and DAPI-positive nuclei were counted using the ImageJ Particle Analyzer after image conversion (threshold function) and image segmentation (watershed function). Regions of interest (ROIs) were segmented based on intensity thresholds of the nuclei and nuclei numbers from segmented ROIs were extracted. The segmentation accuracy was verified manually for three images. The proportion of SOX9-positive to WT1-positive Sertoli cells (SOX9/WT1) was determined by the ratio between SOX9-positive nuclei and WT1-positive nuclei on each sample section.

### Hormone analysis

To evaluate hormone production, media samples were collected every 48 h during the TO culture period and analysed by specific enzyme-linked immunosorbent assays (ELISAs) for testosterone (EIA-1559, AH diagnostics, Solna, Sweden) and AMH (A79765, Beckman Coulter, Brea, CA, USA) production according to the manufacturer’s instructions. The FLUO Star Omega microplate reader (BMG LABTECH, Offenburg, Germany) and MARS Data Analysis software, version 2.10 R3 (BMG LABTECH) were used to measure absorbance. Cubic regression curve fitting was used to determine AMH concentrations. Testosterone concentrations were calculated using a Four-Parameter Logistic curve fit. Duplicates were performed for all samples, calibrators, and controls. AMH concentrations of 0.13 ng/ml and testosterone concentrations of 0.083 ng/ml were assigned a quantitation limit of zero.

### Spermatogonial numbers per round tubules (S/T) and Z-score calculations

For the evaluation of spermatogonial numbers, at least two independent sections of testicular tissue obtained on the day of biopsy, were immunostained against the germ cell marker DDX4, as described above. Spermatogonia were identified based on their morphology (size, shape), location (Paniagua and Nistal, 1984), and DDX4 expression. Employing a blinded approach, all round tubular cross-sections within the tissue sections were quantified and respectively classified (Pampanini *et al.*, 2020); for a comparison of spermatogonial numbers across samples, mean spermatogonial numbers per round tubular cross-section (S/T) were assessed (Supplementary Table S4). To control for physiological variation in spermatogonial numbers during development, age-independent Z-scores were calculated for S/T using the reference means, as recently described (Funke *et al.*, 2021). For statistical analysis, only sections with more than 25 round seminiferous cords were considered.

### Statistical analysis

Statistical analyses of immunofluorescence staining and ELISAs were performed using GraphPad Prism software (GraphPad Prism 7, Boston, MA, USA). All data are presented as the mean and standard deviation (mean ± SD). Statistical significance was considered when the *P*-value was *P* < 0.05. For immunofluorescence staining, the statistical significance between the two groups was evaluated using the Mann–Whitney test. For hormone production quantification by ELISA, statistical significance at the different time points of the two groups was evaluated using the Kruskal–Wallis test. The Pearson correlation coefficient (*r*) was used to study correlations between SOX9-expression and patient age, treatment characteristics and organoid characteristics (Supplementary Table S2). Receiver operating characteristic (ROC) curves and area under the curve (AUC) were used to illustrate the diagnostic value of SOX9 expression in predicting the capacity of testicular tissue to form organoids. Correlation and ROC analyses were performed using IBM SPSS statistical software (version 27, IBM, Chicago, IL, USA).

## Results

### Human TOs can be generated from pre- and peri-pubertal testicular cells

Fresh testicular tissue samples from 11 patients (0.8–13.2 years of age, P1 to P11; Supplementary Table S1) were enzymatically digested into single-cell suspensions and applied to 3-LGS to test their capacity to generate TOs (Fig. 1A). After 7 days in culture, two patterns of reorganization were observed. Primary testicular cells from four patient samples (P3, P4, P5, and P10; Supplementary Table S1) generated single aggregates (Group 1), whereas testicular cells from the other seven patient samples produced multiple aggregates (MAs; Group 2; Fig. 1A and B). The area occupied by single aggregates was significantly larger than the area occupied by each individual aggregate in MAs (*P* < 0.0001; Fig. 1C). In contrast to MAs, single aggregates were classified as TOs after histological analysis revealed compartmentalized seminiferous cord- and interstitial-like structures that closely recapitulated the *in vivo* testicular tissue structure (Fig. 1D, Supplementary Fig. S1).

**Figure 2.**
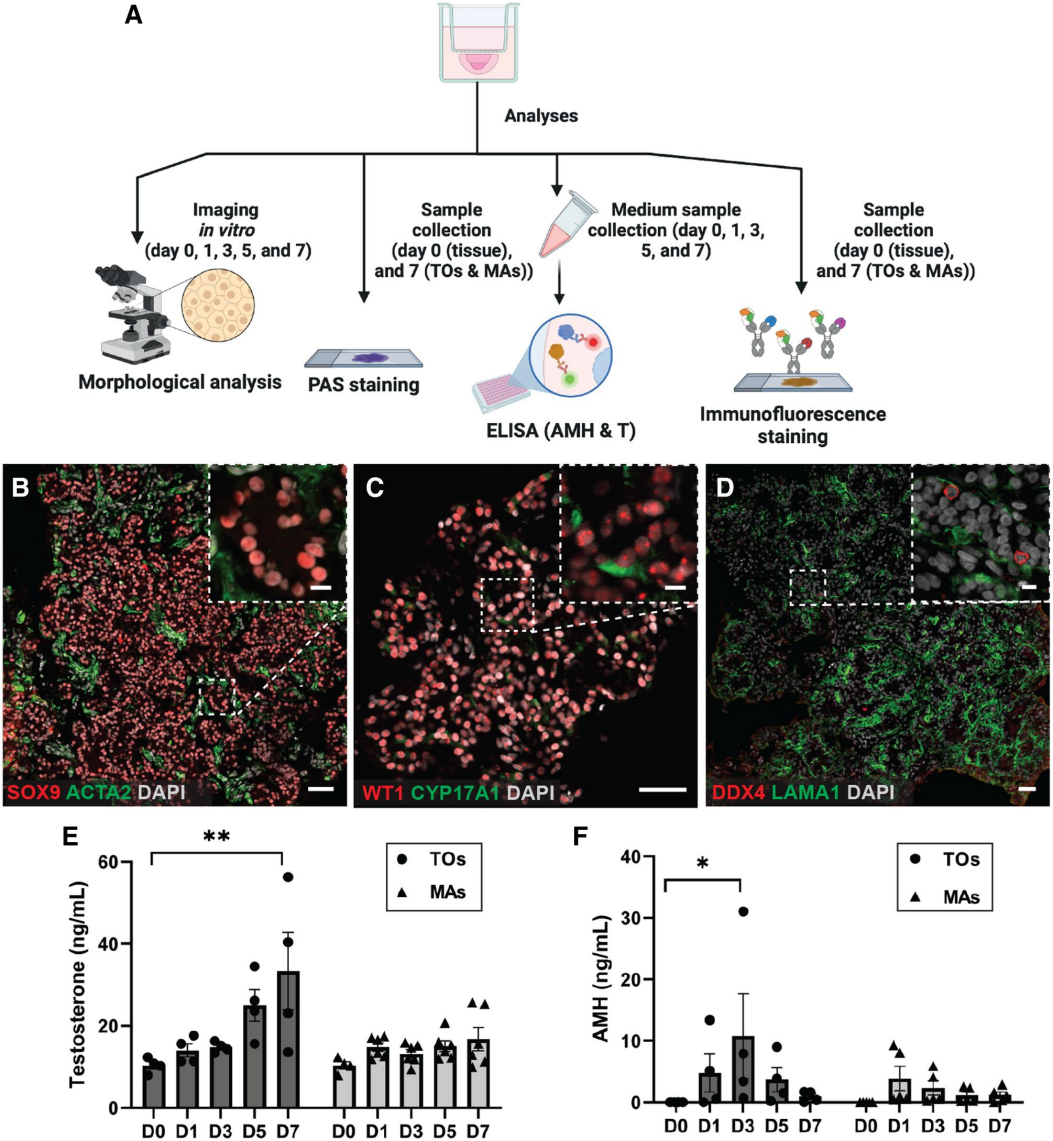
**Characterization of human prepubertal testicular organoids (TOs)**. (**A**) Schematic illustration of the TOs characterization analyses (created with BioRender^®^). (**B**) Immunofluorescent staining of a TO section depicting the localization of the Sertoli cell marker SOX9 (red staining) and the peritubular cell marker ACTA2 (green staining). (**C**) Immunofluorescent staining of a TO section illustrating the localization of the Sertoli cell marker WT1 (red staining) and the Leydig cell marker CYP17A1 (green staining). (**D**) Immunofluorescent staining of a TO section illustrating the localization of germ cell marker DDX4 (red staining) and the basement membrane component LAMA1 (green staining); Scale bars = 50 µm. (**E**) Testosterone (ng/ml) and (**F**) AMH (ng/ml) measurements in the media of TO and multiple aggregates (MAs) cultures. The Kruskal–Wallis test was used to determine the statistical significance among the different time points of the two groups. **P < 0.01, *P < 0.05. SOX9, SRY-Box transcription factor; WT1, Wilms tumour protein 1; DDX4, DEAD-box helicase 4; ACTA2, actin alpha 2; CYP17A1, cytochrome P450 family 17 subfamily A member 1; LAMA1; laminin alpha 1; AMH, anti-Müllerian hormone; PAS, periodic acid–Schiff.

Apart from morphological and histological analysis, we further characterized the cellular composition and hormone production in TO cultures by immunofluorescence staining and ELISA, respectively (Fig. 2A).

### Cellular composition and organization of TOs resemble features of human prepubertal testicular architecture

Immunohistochemical analysis of TOs showed cells expressing the Sertoli cell markers SOX9 and WT1 inside the cord-like structures (Fig. 2B and C, respectively). Moreover, the cord-like structures were surrounded by cells expressing the peritubular cell marker ACTA2 and LAMA1, a basement membrane component (Fig. 2B and D, respectively). A small number of DDX4-positive germ cells were detected inside the cord-like structures (Fig. 2D, Supplementary Table S4). Cells expressing the Leydig cell marker CYP17A1 were detected in the interstitial-like compartment of three out of four TO cultures (Fig. 2C). In contrast, incorrect localization of SOX9 and CYP17A1 expressing cells, and no DDX4-positive germ cells were observed in MA cultures (Supplementary Fig. S2).

### TOs produce testosterone and AMH

In immature testes, Sertoli cells produce AMH, but expression declines with maturation (Stukenborg *et al.*, 2010). In contrast, testosterone is produced by Leydig cells during embryonic and foetal life and its secretion is reinitiated at the beginning of puberty (Stukenborg *et al.*, 2010). Accordingly, TOs and MAs both produced testosterone and AMH throughout the 7-day culture period. The testosterone concentration in the TO culture media increased significantly (*P* < 0.05) from day zero to day seven (Fig. 2E). Moreover, the AMH concentration in the TOs culture media increased from day zero to day three before declining during the remaining culture period (Fig. 2F). On the other hand, MAs exhibited lower secretion of testosterone and AMH to the media over the entire culture period (Fig. 2E and F).

### Prepubertal to formation correlates with expression of SOX9

**Figure 3.**
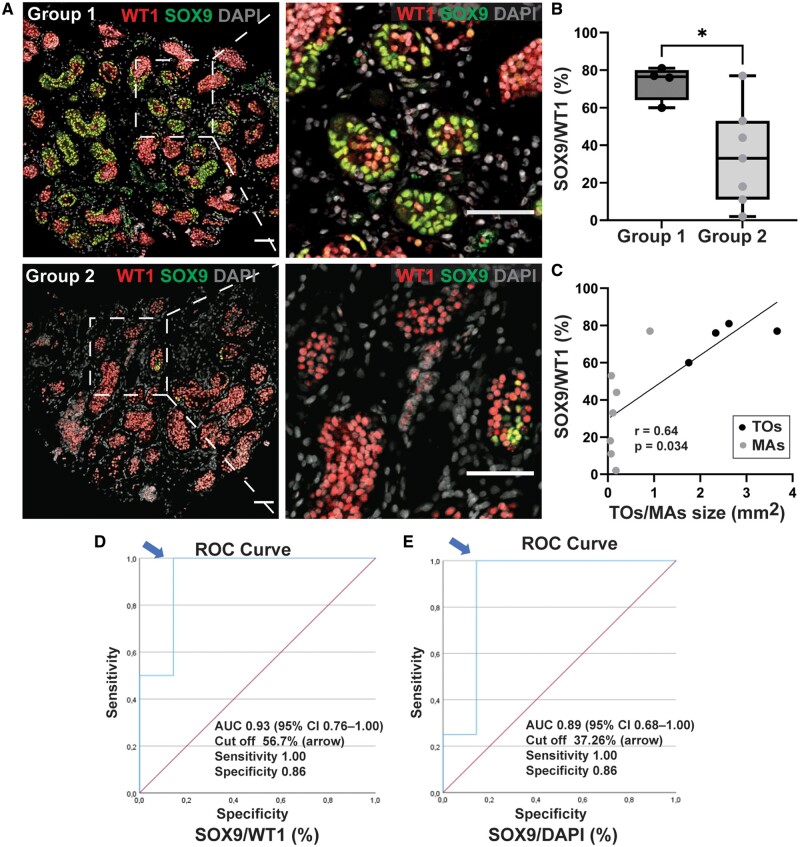
**SOX9 expression in prepubertal testicular tissue correlates with its capacity for organoid formation**. (**A**) Representative immunofluorescence staining images for SOX9 (green), WT1 (red), and nuclear marker DAPI (grey) in testicular samples. Scale bar = 100 µm. (**B**) The proportions of SOX9-positive and WT1-positive Sertoli cells (SOX9/WT1) for samples with (Group 1) or without (Group 2) the capacity to form organoids, *P = 0.0273. (**C**) Correlation between aggregate/organoid size and proportion of SOX9-expressing Sertoli cells; *r* = 0.064, *P* = 0.034. (**D**) Receiver operating characteristic (ROC) curves for predicting the capacity for organoid formation based on the proportion of SOX9-expressing nuclear area of Sertoli cells. (**E**) the proportion of SOX9-expressing cells per cross-section (SOX9/DAPI). Figures present areas under the curve (AUC), and arrows depict cut-off points with good prognostic values and corresponding sensitivities and specificities. SOX9, SRY-Box transcription factor 9; WT1, Wilms tumour protein 1; TO, testicular organoids; MAs, multiple aggregates.

Sertoli cells are known to orchestrate cellular reorganization during TO formation (Alves-Lopes *et al.*, 2017, 2018; Oliver *et al.*, 2021). To investigate the potential correlations between the expression of Sertoli cell markers and the capacity to form TOs, immunofluorescence analysis against WT1 and SOX9 was performed on the prepubertal testicular samples utilized to test organoid formation (Fig. 3A). Notably, the expression of SOX9 was variable among the 11 testicular samples analysed (Fig. 3A; Supplementary Table S2). This expression pattern was confirmed with a second antibody against SOX9 (Supplementary Fig. S3). Importantly, virtually all Sertoli cells, including those that were SOX9-negative, expressed the marker WT1, allowing the determination of the Sertoli cell fraction expressing SOX9. Remarkably, the proportion of SOX9-expressing Sertoli cells was significantly higher (73.5 ± 0.093% vs 34.0 ± 26.2%; *P* = 0.027) in testicular samples, which formed TOs compared to those that generated MAs (Fig. 3B and C). Moreover, in our ROC curve analysis, a cut-off of 56.70% for SOX9/WT1 and 37.26% for SOX9/DAPI in testicular tissue had good prognostic value (AUC: 0.93, and 0.89, respectively) and was highly predictive of TO formation (Fig. 3D and E).

Additionally, the expression of CYP17A1, ACTA2, or WT1 could be observed in the testicular tissue samples that formed TOs and MAs (Supplementary Fig. S3, Supplementary Table S4). Furthermore, the expression of the Sertoli cell maturation marker GATA1 or AR was not observed in the seminiferous cords from all 11 testicular tissue samples analysed; however, AR could be detected in the interstitial compartment of 9 out of 11 samples at Day 0 (Supplementary Fig. S3, Supplementary Table S4). Regarding germ cells, the number of DDX4-positive cells per round seminiferous cords (S/T) were counted and evaluated in the 11 testicular samples used in these experiments. By applying in the analysis Z-scores, which indicate if the S/T-values are within the normal range (±3 SD) from the mean and SD of the respective age groups, the results revealed that germ cells are not crucial for TO formation (Supplementary Table S4).

### Decreased SOX9 expression in testicular samples collected for fertility preservation correlates with higher exposure to alkylating agents

**Figure 4.**
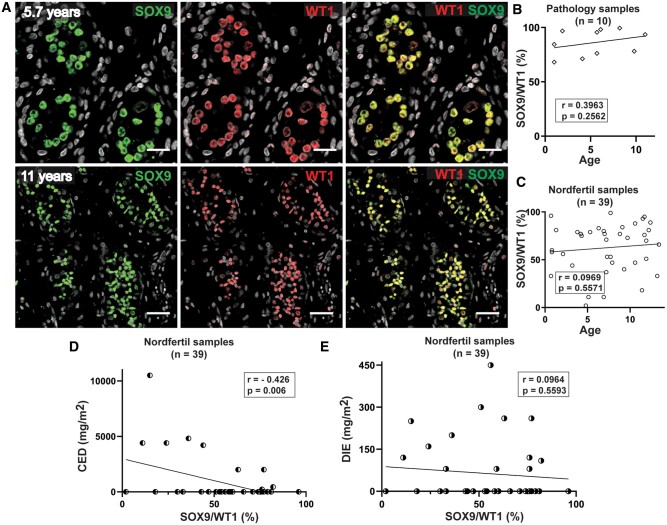
**SOX9 expression in testicular samples from sample collections of the pathological biobank (n = 10) and NORDFERTIL sample collection (n = 39)**. (**A**) Representative immunofluorescence staining images for Sertoli cells marker SOX9 and WT1 in biobank samples. Scale bar = 50 µm. (**B** and **C**) Proportion of SOX9-expressing Sertoli cells showed no correlation with patient age in the sample collection of the pathological biobank (B) or NORDFERTIL sample collection (C). (**D** and **E**) Decreased proportions of SOX9-expressing Sertoli cells correlated with increased exposure to alkylating agents (cyclophosphamide equivalent dose (CED) (*r* = −0.426, *P* = 0.006)) (D) but not with exposure to anthracyclines (doxorubicin isotoxic dose equivalents (DIE) (*r* = −0.096, *P* = 0.559)) (E). SOX9, SRY-Box transcription factor 9; WT1, Wilms tumour protein 1; *r*, Pearson correlation coefficient.

To further characterize the patterns of SOX9 expression in pre- and peri-pubertal testicular tissue, immunofluorescence staining against SOX9 and WT1 was performed in additional 28 testicular samples from the NORDFERTIL sample collection, including a wide range of primary diagnoses and treatment exposures (aged 6.9 ± 3.8 (mean ± SD) years, P12 to P39, Supplementary Table S1), and in 10 samples from the sample collection of the Pathology Department without known testicular disease (aged 5.5 ± 3.5 (mean ± SD) years, P40–P49, Supplementary Table S1). The majority of Sertoli cells (SOX9/WT1 86.1 ± 12.1% (mean ± SD)) in the sample collection of the Pathology Department co-expressed SOX9 and WT1 (Fig. 4A). Moreover, no correlation was observed between the proportion of SOX9-positive cells (SOX9/WT1) and age in both sample collection sets (Fig. 4B and C). Notably, a decreased proportion of SOX9-expressing Sertoli cells was correlated with increased exposure to alkylating agents (CED) (*r* = −0.426, *P* = 0.006), but not to anthracycline chemotherapy, i.e. DIE (*r* = −0.096, *P* = 0.559) in the testicular tissues from the NORDFERTIL sample collection (n = 39; Fig. 4D and E, Supplementary Table S2).

## Discussion

In this study, we showed for the first time that human pre- and peri-pubertal testicular cells can successfully generate TOs with cord-like structures and an interstitial-like compartment. These organoids can secrete testosterone and AMH during a 7-day culture period. Our study also revealed a significant variation in SOX9 expression in prepubertal testicular tissues collected for fertility preservation. A high proportion (≥50%) of SOX9-expressing Sertoli cells predicted TO formation capacity. Furthermore, we discovered that higher exposure to alkylating agents was negatively correlated with the proportion of Sertoli cells expressing SOX9 in prepubertal testicular tissue.

Previously, our group generated TOs using testicular cells from postnatal rats as well as cells from embryonic human testes and ovaries (Alves-Lopes *et al.*, 2017, 2018; Oliver *et al.*, 2021). While human embryonic ovarian organoids can support germ cell survival, this was not the case in human embryonic TOs, where germ cells were lost (Oliver *et al.*, 2021). In the present study, DDX4-positive germ cells were observed in prepubertal TOs in one out of four patients after 7 days of culture. Although prepubertal TOs can support germ cells, further optimization of the culture conditions is required to improve germ cell maintenance and to further differentiate germ cells.

A first study on TOs showed that cryopreserved human adult and pubertal testicular cells can self-organize into multi-cellular aggregates, with or without a biological scaffold (Baert *et al.*, 2017). However, although showing some features of testicular cells, such as testosterone and inhibin B production, these aggregates lacked specific testis topography. In agreement with these results, testosterone production and early germ cell differentiation were observed, when using cryopreserved adult human testicular cell suspensions to form TOs in culture conditions supplemented with solubilized human testicular extracellular matrix (Pendergraft *et al.*, 2017). Further, the potential of TOs, generated from fresh and cryopreserved tissue samples, and their use in high-throughput studies, was explored by culture conditions using microwells (Sakib *et al.*, 2019a,b). Those generated TOs, from fresh murine or pig cells, as well as cryopreserved primate testicular cells, contained all of the major testicular cell types but revealed an inside-out organization, which differs from the testicular cord structures present *in vivo*.

The patient-derived TOs created in this study provide a model for studying testicular somatic cell function and the effects of the testicular microenvironment on germ cell survival, because they recapitulate the *in vivo* testicular architecture of cord-like structures. By providing a novel tool to monitor testicular somatic cell functions (e.g. hormone production, generation of blood–testis barrier formation, and vascularization), human TOs might offer new insights into the needs for clinical fertility preservation, such as germ cell and testicular tissue transplantation assays, which often depend on functional somatic cells.

This study demonstrates that 7 out of 11 primary testicular cells derived from the fresh testicular tissue biopsy from prepubertal boys were not capable of forming TOs, which is a putative indicator of affected testicular somatic components in these samples. Our observations highlight the impact of cancer treatment on somatic cell populations in the testis and how this can influence the regenerative potential of seminiferous tubules for fertility preservation. Further studies are needed to reveal the mechanisms by which alkylating agents affect SOX9 expression in prepubertal testes. In addition to alkylating agents, other drugs and chemicals (e.g. endocrine-disrupting compounds) may also impair testicular somatic cell function and SOX9 expression, which may be uncovered by perturbations in TO formation.

Previous studies using rat and mouse testicular tissue suggested that the ability to form TOs is age-dependent, with increased capacity associated with younger age, independent of the culture microenvironment (Alves-Lopes *et al.*, 2017; Edmonds and Woodruff, 2020). SOX9 expression in Sertoli cells of mice, rats, and dogs has been shown to be age- and spermatogenetic-stage-dependent, with decreasing expression in rodent models with increasing age (Frojdman *et al.*, 2000; Aksel *et al.*, 2023). In this study, the results in humans showed that SOX9 expression in the NORDFERTIL sample collection and the sample collection from the Pathology Department did not show age dependency. Moreover, pre- and peri-pubertal TO formation was not correlated with the age of the patient, but rather with SOX9 expression in the Sertoli cells.

The role of SOX9 in TO formation has not been investigated previously, and the precise mechanisms involved remain unknown. Interestingly, SOX9 expression has also been detected in other organs, including the bone, heart, lung, pancreas, intestine, and nervous system (Ming *et al.*, 2022). Similar to the present observations, the inactivation of SOX9 expression has been shown to decrease the proliferation and differentiation of human lung organoids (Li *et al.*, 2021).

However, despite finding that TO formation positively correlates with SOX9 expression in Sertoli cells, which in turn negatively correlates with previous exposure to alkylating chemotherapy, the limited amount of material available allowed us to use only 11 out of the 39 prepubertal testicular tissue samples for the organoid formation experiments. It is crucial to confirm this correlation in a larger sample size to ensure the robustness and generalizability of our results. A more extensive dataset would provide greater statistical power and help validate the observed relationship.

Additionally, when considering the application of our findings to organoid cultures derived from frozen–thawed testicular tissue samples, it is important to evaluate whether the cryopreservation process affects the expression of SOX9. The viability and functionality of testicular cells post-thaw could influence the efficiency of organoid assembly and, consequently, the applicability of our correlation in different experimental settings.

In conclusion, the expression of SOX9 in prepubertal testicular tissue was positively correlated with its capacity to form TOs and negatively correlated with exposure to alkylating agents. Our observations suggest that SOX9-expression, in addition to hormone measurements, may serve as an indicator to estimate additional aspects of testicular tissue samples from childhood cancer patients. In addition, patient-derived TOs might serve as a novel tool to study mechanisms related to testicular morphogenesis, the SSC niche, and the effects of genetic diseases and drug toxicity, guiding the optimal use of cryopreserved testicular tissue in future fertility preservation strategies.

## Supplementary Material

hoae049_Supplementary_Data

## Data Availability

The raw data included in this study are available upon request from the corresponding author.
