# Supplementary material for: Prior exposure to alkylating agents negatively impacts testicular organoid formation in cells obtained from childhood cancer patients
Source: Hum Reprod Open. 2024 Aug 13;2024(3):hoae049. doi: 10.1093/hropen/hoae049 (PMC11346771; doi:10.1093/hropen/hoae049)
Supplement: hoae049_Supplementary_Data [file hoae049_supplementary_data.zip › Supplementary Figure S3_new_20240606.pdf]

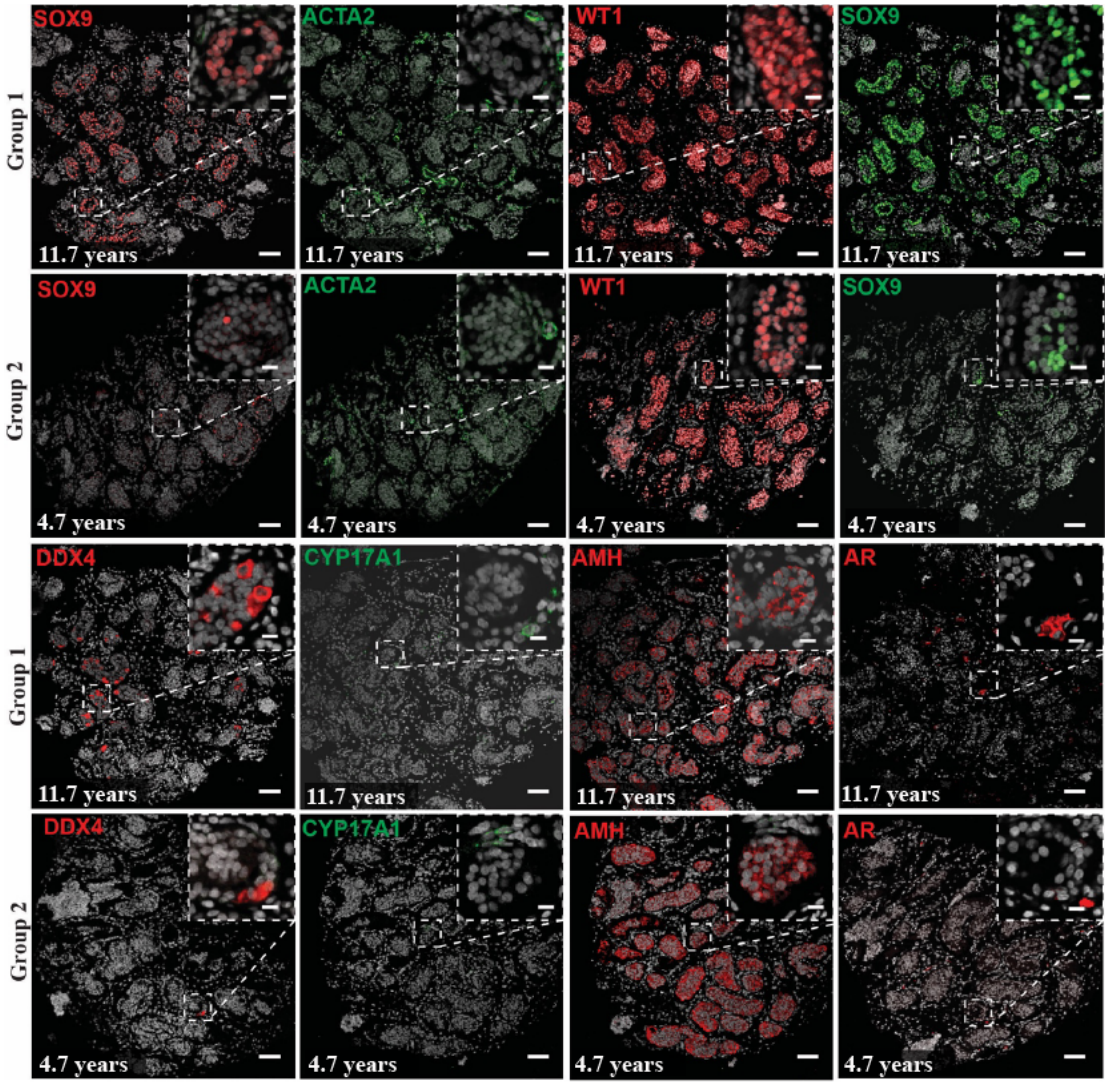

Supplementary Figure S3: Expression of testicular cell markers in representative prepubertal testicular tissue samples of two patients grouped by their capacity to testicular organoid assembly

Expression of Sertoli cells marker SOX9 and WT1, peritubular cells marker ACTA2, germ cells marker DDX4, and Leydig cells marker CYP17A1 in testicular tissue with (P4, 11.7 years old, group 1) or without (P7, 4.7 years old, group 2) capacity for testicular organoid formation. Scale bar = 50 µm (insets, 10 µm).
