# Supplementary material for: Prior exposure to alkylating agents negatively impacts testicular organoid formation in cells obtained from childhood cancer patients
Source: Hum Reprod Open. 2024 Aug 13;2024(3):hoae049. doi: 10.1093/hropen/hoae049 (PMC11346771; doi:10.1093/hropen/hoae049)
Supplement: hoae049_Supplementary_Data [file hoae049_supplementary_data.zip › Supplementary Table S1 - 20240715 R2.docx]

**Supplementary Table S1: Patients and respective testicular samples information.**

| **Patients** | **Age/y** | **Diagnosis** | **CED mg/m^2^** | **DIE mg/m^2^** | **SOX9/WT1**  **(%)** | **Organoid formation (Y/N/NT)** |
| --- | --- | --- | --- | --- | --- | --- |
| P1 | 6.9 | Leukaemia | 4400 | 120 | 11.1% | N |
| P2 | 3.2 | Solid tumour | 4200 | 0 | 44.3% | N |
| P3 | 8.8 | Leukaemia | 2000 | 260 | 77.4% | Y |
| P4 | 11.7 | Leukaemia | 220 | 120 | 75.7% | Y |
| P5 | 0.8 | Immunodeficiency | 0 | 0 | 60.2% | Y |
| P6 | 4.2 | Hypogonadism | 0 | 0 | 77.1% | N |
| P7 | 4.8 | Immunodeficiency | 0 | 0 | 1.7% | N |
| P8 | 7.7 | Non-malignant haematological disease | 0 | 0 | 53.2% | N |
| P9 | 11.4 | Immunodeficiency | 0 | 0 | 17.5% | N |
| P10 | 12.2 | Immunodeficiency | 0 | 0 | 80.8% | Y |
| P11 | 13.2 | Non-malignant haematological disease | 0 | 0 | 33.3% | N |
| P12 | 1.3 | Solid tumour | 10500 | 250 | 15.2% | NT |
| P13 | 6.3 | Solid tumour | 4814 | 200 | 36.2% | NT |
| P14 | 6.9 | Leukaemia | 4400 | 160 | 24.2% | NT |
| P15 | 9.7 | Leukaemia | 2000 | 260 | 63.0% | NT |
| P16 | 6.7 | Leukaemia | 448 | 109 | 81.7% | NT |
| P17 | 2.1 | Solid tumour | 0 | 450 | 56.4% | NT |
| P18 | 11.9 | Leukaemia | 0 | 300 | 50.7% | NT |
| P19 | 9.2 | Non-malignant haematological disease | 0 | 80 | 59.1% | NT |
| P20 | 10.6 | Leukaemia | 0 | 80 | 75.9% | NT |
| P21 | 11.9 | Leukaemia | 0 | 80 | 33.0% | NT |
| P22 | 0.7 | Leukaemia | 0 | 0 | 96.1% | NT |
| P23 | 0.7 | Leukaemia | 0 | 0 | 76.4% | NT |
| P24 | 0.8 | Leukaemia | 0 | 0 | 57.9% | NT |
| P25 | 3.0 | Immunodeficiency | 0 | 0 | 80.6% | NT |
| P26 | 3.8 | Leukaemia | 0 | 0 | 42.6% | NT |
| P27 | 4.3 | Non-malignant haematological disease | 0 | 0 | 32.9% | NT |
| P28 | 5.1 | Non-malignant haematological disease | 0 | 0 | 30.0% | NT |
| P29 | 5.3 | Metabolic syndrome | 0 | 0 | 79.2% | NT |
| P30 | 7.0 | Non-malignant haematological disease | 0 | 0 | 42.9% | NT |
| P31 | 7.2 | Non-malignant haematological disease | 0 | 0 | 71.0% | NT |
| P32 | 7.3 | Leukaemia | 0 | 0 | 53% | NT |
| P33 | 7.7 | Non-malignant haematological disease | 0 | 0 | 53.8% | NT |
| P34 | 7.9 | Non-malignant haematological disease | 0 | 0 | 47.5% | NT |
| P35 | 9.3 | Non-malignant haematological disease | 0 | 0 | 80.9% | NT |
| P36 | 10.6 | Non-malignant haematological disease | 0 | 0 | 53.3% | NT |
| P37 | 11.5 | Non-malignant haematological disease | 0 | 0 | 74.4% | NT |
| P38 | 11.7 | Leukaemia | 0 | 0 | 46.9% | NT |
| P39 | 13.4 | Immunodeficiency | 0 | 0 | 66.3% | NT |
| P40 | 1.0 | Pathology biobank sample collection | 0 | 0 | 84.4% | NT |
| P41 | 1.0 | Pathology biobank sample collection | 0 | 0 | 68.2% | NT |
| P42 | 1.9 | Pathology biobank sample collection | 0 | 0 | 96.9% | NT |
| P43 | 4.1 | Pathology biobank sample collection | 0 | 0 | 71.2% | NT |
| P44 | 5.7 | Pathology biobank sample collection | 0 | 0 | 76.2% | NT |
| P45 | 5.7 | Pathology biobank sample collection | 0 | 0 | 95.6% | NT |
| P46 | 6.1 | Pathology biobank sample collection | 0 | 0 | 98.2% | NT |
| P47 | 8.2 | Pathology biobank sample collection | 0 | 0 | 99.4% | NT |
| P48 | 9.8 | Pathology biobank sample collection | 0 | 0 | 78.1% | NT |
| P49 | 11.0 | Pathology biobank sample collection | 0 | 0 | 93.5% | NT |

The table contains information regarding the age in years, the cumulative cyclophosphamide equivalent dose (CED) in mg/m^2^, the doxorubicin isotoxic dose equivalent (DIE) in mg/m^2^, the ratio between SOX9-positive and WT1-positive Sertoli cells in percentage, and the capacity for testicular organoid formation for the 49 patients (P) included in this study. Only eleven samples (P1-P11) from the 39 collected under the scope of the NORDFERTIL fertility preservation program were tested for testicular organoid formation, due to restrictions in the amount of tissue collected from the remaining 28 patients. Additionally, ten samples from the sample collection of the pathology biobank (P40-P49) without known testicular pathology were also included. Abbreviations: Y, yes; N, no; NT, not tested.
