# Supplementary material for: Prior exposure to alkylating agents negatively impacts testicular organoid formation in cells obtained from childhood cancer patients
Source: Hum Reprod Open. 2024 Aug 13;2024(3):hoae049. doi: 10.1093/hropen/hoae049 (PMC11346771; doi:10.1093/hropen/hoae049)
Supplement: hoae049_Supplementary_Data [file hoae049_supplementary_data.zip › Supplementary Table S2 - 20240715 R2.docx]

**Supplementary Table S2. Patient and sample information.**

| **Parameter** | **Variable** | **Cultured samples n=11** | | **Archived samples n=28** | |
| --- | --- | --- | --- | --- | --- |
|  |  | **r** | **P** | **r** | **P** |
| SOX9/WT1 % |  |  |  |  |  |
|  | Age/y | 0.76 | 0.82 | -0.01 | 0.95 |
|  | Chemotherapy exposure/ yes/no | 0.10 | 0.77 | -0.35 | 0.069 |
|  | CED/ mg/m^2^ | -0.12 | 0.71 | **-0.452** | **0.004** |
|  | DIE/ mg/m^2^ | 0.24 | 0.47 | -0.33 | 0.09 |
|  | Z-score/SD | -0.06 | 0,87 | 0.46 | **0.013** |
|  | Organoid assembly/ yes/no | 0.69 | **0.019** |  |  |
|  | SOX9/DAPI/% | 0.95 | **<0.001** |  |  |
|  | Aggregate/culture dish area/mm^2^ | 0.64 | **0.034** |  |  |
| SOX9/DAPI % |  |  |  |  |  |
|  | Age/y | 0.08 | 0.82 | 0.09 | 0.63 |
|  | Chemotherapy exposure/ yes/no | 0.07 | 0.84 | -0.31 | 0.11 |
|  | CED | -0.12 | 0.72 | -0.41 | **0.032** |
|  | DIE | 0.20 | 0.57 | -0.20 | 0.31 |
|  | Z-score/SD | -0.06 | 0.86 | 0.44 | **0.02** |
|  | Organoid assembly/ yes/no | 0.75 | **0.008** |  |  |
|  | Aggregate/culture dish area/ mm^2^ | 0.64 | **0.043** |  |  |
| Aggregate/culture dish area/mm^2^ |  |  |  |  |  |
|  | Age/y | 0.10 | 0.77 |  |  |
|  | Chemotherapy exposure/ yes/no | 0.33 | 0.32 |  |  |
|  | CED | 0.04 | 0.92 |  |  |
|  | DIE | 0.51 | 0.11 |  |  |
|  | Z-score/SD | -0.16 | 0.64 |  |  |
|  | Organoid assembly/ yes/no | 0.95 | **<0.001** |  |  |

The table contains information regarding the correlations of patient’s age (years), treatment characteristics and organoid characteristics with the ratio between SOX9-positive and WT1-positive Sertoli cells in percentage (SOX9/WT1), the ratio between SOX9-positive and DAPI-positive cells in percentage (SOX9/DAPI) and area of organoid/aggregation in the eleven cultured samples and 28 archived NORDFERTIL samples only analysed by histology. Abbreviations: standard deviation (SD), cumulative cyclophosphamide equivalent dose (CED), doxorubicin isotoxic dose equivalent (DIE), Pearson correlation coefficient (r)
