# Supplementary material for: Prior exposure to alkylating agents negatively impacts testicular organoid formation in cells obtained from childhood cancer patients
Source: Hum Reprod Open. 2024 Aug 13;2024(3):hoae049. doi: 10.1093/hropen/hoae049 (PMC11346771; doi:10.1093/hropen/hoae049)
Supplement: hoae049_Supplementary_Data [file hoae049_supplementary_data.zip › Supplementary Table S3 - 20240715 R2.docx]

**Supplementary Table S3: Primary and secondary antibodies.**

| **Protein** | **Host**  **Species** | **Antibody concentration** | **Antibody dilution** | **Catalogue number** | **Source** | **Main localization** |
| --- | --- | --- | --- | --- | --- | --- |
| SOX9 | mouse | 0.5 mg/mL | 1:400 | ab76997 | Abcam (Cambridge, UK) | Sertoli cells (Nuclear) |
| SOX9 | rabbit | 1 mg/mL | 1:400 | ab5535 | Sigma-Aldrich, (Saint Louis, MO, USA) | Sertoli cells (Nuclear) |
| WT1 | rabbit | 0.221 mg/mL | 1:200 | ab89901 | Abcam | Sertoli cells (Nuclear) |
| DDX4 | rabbit | 1 mg/mL | 1:200 | ab27591 | Abcam | Germ cells (Cytoplasm) |
| ACTA2 | mouse | 6.0 mg/mL | 1:500 | a2547 | Sigma-Aldrich | Peritubular cells (Cytoplasm) |
| CYP17A1 | mouse | 200 µg/ml | 1:200 | sc374244 | Santa Cruz Biotechnology (Dallas, TX, USA) | Leydig cells (Cytoplasm) |
| LAMA1 | mouse | 0.7 mg/mL | 1:100 | ab210954 | Abcam | ECM |
| AMH | goat | 1mg/mL | 1:100 | af2748 | R&D Systems (Minneapolis, MN, USA) | Sertoli cells (Cytoplasm) |
| AR | rabbit | 0.49 mg/m | 1:100 | ab108341 | Abcam | Leydig cells (Nuclear) |
| GATA1 | rabbit | 1.04 mg/ml | 1:100 | ab181544 | Abcam | Sertoli cells (Nuclear) |
| Mouse IgG | mouse | 0.4 mg/ml | 1:100 | sc-2025 | Santa Cruz Biotechnology | N/A |
| Rabbit IgG | rabbit | 1.775 mg/ml | 1:200 | ab172730 | Abcam | N/A |
| Goat IgG | goat | 1 mg/ml | 1:200 | AB-108-C | R&D Systems | N/A |
| Cy3 | rabbit | 1.5 mg/ml | 1:500 | 711-166-152 | Jackson ImmunoResearch (West Grove, PA, USA) | N/A |
| Alexa Fluor 488 | mouse | 1.5 mg/ml | 1:500 | 715-546-150 | Jackson ImmunoResearch | N/A |

The table summarizes the key features, sources, and applications of primary and secondary antibodies in immunofluorescence staining based on information provided by the human protein atlas (<https://www.proteinatlas.org/>). Abbreviations: SRY-Box transcription factor 9 (SOX9), Wilms tumour protein 1 (WT1), DEAD-box helicase 4 (DDX4), actin alpha 2 (ACTA2), cytochrome P450 family 17 subfamily A member 1 (CYP17A1), laminin alpha 1 (LAMA1), anti-Müllerian hormone (AMH), androgen receptor (AR), GATA binding protein 1 (GATA1), Cyanine Dye 3 (Cy3)
