# Supplementary material for: Prior exposure to alkylating agents negatively impacts testicular organoid formation in cells obtained from childhood cancer patients
Source: Hum Reprod Open. 2024 Aug 13;2024(3):hoae049. doi: 10.1093/hropen/hoae049 (PMC11346771; doi:10.1093/hropen/hoae049)
Supplement: hoae049_Supplementary_Data [file hoae049_supplementary_data.zip › Supplementary Table S4 - 20240715 R2.docx]

**Supplementary Table S4: Information of samples tested for organoid formation experiments.**

| **Patients** | **Germ cells day 0 (S/T)** | **Z-scores** | **Number of cell aggregates** | **Mean area of aggregates (mm^2^)** | **Number of cords** | **DDX4 pos. cells/ cord** | **CYP17A1 pos. cells/ mm^2^** | **AR pos. cells/ mm^2^** |
| --- | --- | --- | --- | --- | --- | --- | --- | --- |
| P1 | 0.05 | -18.3 | 31 | 0.01 | 0 | 0 | 12.90 | 0.00 |
| P2 | 0.76 | -3.1 | 18 | 0.06 | 0 | 0 | 64.03 | 44.82 |
| P3 | 0.06 | -23.4 | 1 | 1.20 | 23 | 0 | 102.23 | 63.29 |
| P4 | 0.56 | -3.2 | 1 | 1.76 | 13 | 0 | 35.10 | 17.55 |
| P5 | 0.51 | -3.4 | 1 | 1.29 | 22 | 0.09 | 0.00 | 143.11 |
| P6 | 0.32 | -6.0 | 14 | 0.03 | 0 | 0 | 3.97 | 8.39 |
| P7 | 0.68 | -3.4 | 14 | 0.07 | 0 | 0 | 25.18 | 39.57 |
| P8 | 1.10 | -2.4 | 15 | 0.05 | 0 | 0 | 0.00 | 17.48 |
| P9 | 0.44 | -3.6 | 17 | 0.04 | 0 | 0 | 100.85 | 0.00 |
| P10 | 0.67 | -2.9 | 1 | 0.81 | 27 | 0 | 28.52 | 69.26 |
| P11 | 4.00 | -0.2 | 29 | 0.01 | 0 | 0 | 7.46 | 21.91 |

The table includes information regarding the germ cell numbers per round seminiferous cord (S/T), the z-scores, and the number of cells expressing CYP17A1 and AR per mm^2^ for each patient at the day of biopsy (day 0). Furthermore, it shows the number and mean area (mm^2^) of the cell aggregates, as well as number of formed cords for the eleven cultured patient samples after seven days in culture. Abbreviations: DEAD-box helicase 4 (DDX4), cytochrome P450 family 17 subfamily A member 1 (CYP17A1), androgen receptor (AR).
