# Supplementary material for: Prior exposure to alkylating agents negatively impacts testicular organoid formation in cells obtained from childhood cancer patients
Source: Hum Reprod Open. 2024 Aug 13;2024(3):hoae049. doi: 10.1093/hropen/hoae049 (PMC11346771; doi:10.1093/hropen/hoae049)
Supplement: hoae049_Supplementary_Data [file hoae049_supplementary_data.zip › Supplementary Figure S1_new_20250530.pdf]

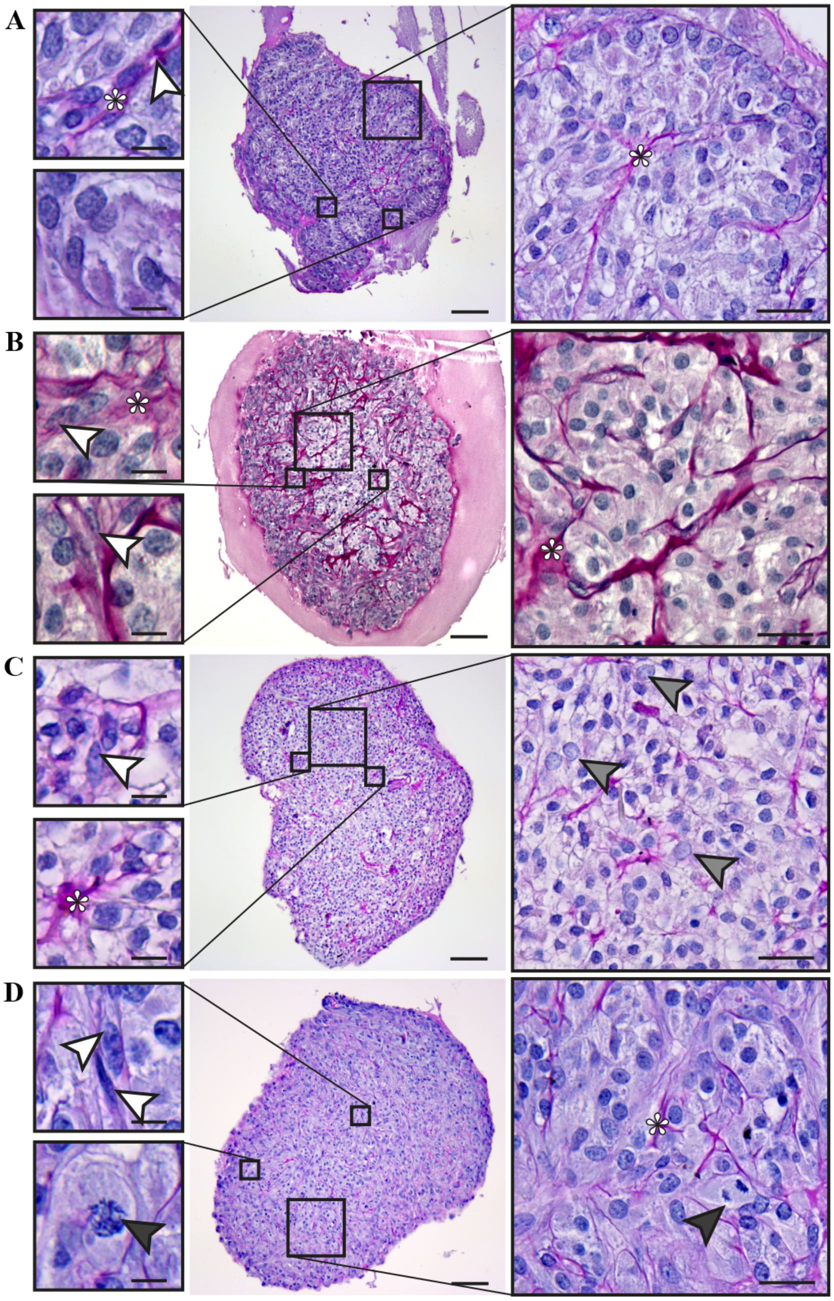

Supplementary Figure S1: Histological evaluation of testicular organoids (TOs) formed form dissociated cell suspensions obtained from prepubertal testicular tissue samples of four patients (P3, 4, 5, and 10).

PAS staining images showing the histology of testicular organoids formed from testicular tissue samples of patients from Group 1 (A, P5, B, P4, C, P10, and D, P3). All TOs showing cord-like structures and interstitial-like compartments. White star: extra cellular matrix, white arrow heads: peritubular cells; light

grey arrowhead: intra-tubular cells; dark grey arrowheads: dividing cells; Scale bars = 100 μm (insets, 10 μm)
