# Supplementary material for: Prior exposure to alkylating agents negatively impacts testicular organoid formation in cells obtained from childhood cancer patients
Source: Hum Reprod Open. 2024 Aug 13;2024(3):hoae049. doi: 10.1093/hropen/hoae049 (PMC11346771; doi:10.1093/hropen/hoae049)
Supplement: hoae049_Supplementary_Data [file hoae049_supplementary_data.zip › Supplementary Figure S2 -20240603.pdf]

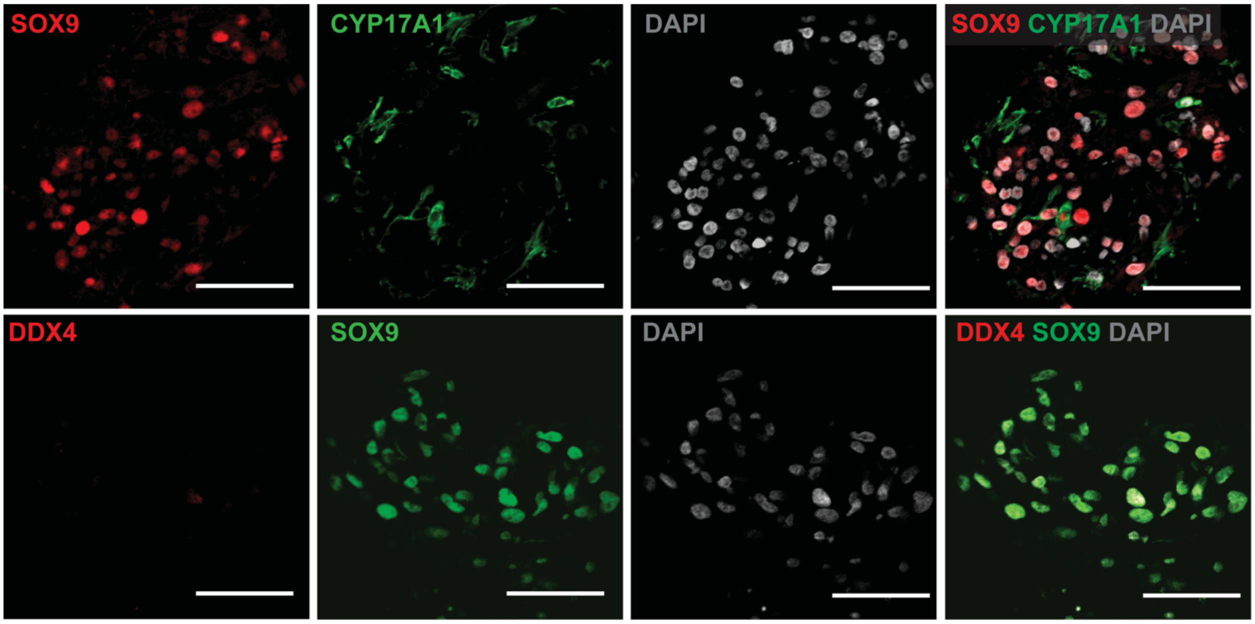


**Supplementary Figure S2: Expression of testicular cell markers in small multiple aggregates without testicular organization after 7 days of culture.**

Defined tubular structures or interstitial compartments were not observed in small multiple aggregates (MAs). Expression of SOX9 (Sertoli cell marker; rabbit anti-SOX9, red staining in the top panel; mouse anti-SOX9, green staining in the lower panel) and CYP17A1 (Leydig cell marker; green staining) was observed, but randomly distributed in the aggregates. DDX4 expression (germ cell marker; potential red staining) was not observed. Scale bars = 50 µm.
